# Pseudoaneurysm following Two-Stage Hip Revision with Fasciotomy

**DOI:** 10.1155/2022/6254542

**Published:** 2022-12-26

**Authors:** Jordan R. Pollock, Kade S. McQuivey, Collin L. Braithwaite, Jennifer Swanson, Joshua S. Bingham

**Affiliations:** ^1^Mayo Clinic Alix School of Medicine, Scottsdale, AZ, USA; ^2^Department of Orthopedics, Mayo Clinic in Arizona, Phoenix AZ, USA; ^3^Oakland University William Beaumont School of Medicine, Rochester Hills, MI, USA

## Abstract

In the setting of total hip arthroplasty (THA), pseudoaneurysms are extremely rare and can be difficult to diagnose, as clinical symptoms can mimic symptoms of other more common complications, such as periprosthetic joint infection, hematoma, and nerve damage. We present a case of a 69-year-old male with a history of slipped capital femoral epiphysis 56 years prior and subsequent right THA. The right hip primary arthroplasty was subsequently complicated by multiple dislocations and recurrent prosthetic joint infections. The most recent infection was treated with debridement, antibiotics, and implant retention (DAIR) in 2017. The patient later presented in 2019 with right thigh pain. Upon further analysis, he was diagnosed with *Streptococcus bovis* positive periprosthetic joint infection. The patient underwent a two-stage revision of the hip using an antibiotic spacer. Two weeks following the second stage, he presented with a sudden onset of uncontrolled atrial fibrillation with rapid ventricular response and a low hemoglobin. The computed tomography scan revealed a large hematoma involving both the anterior and posterior thigh compartments with lab markers that were questionable for infection. An operation to remove the hematoma revealed no purulence, and a large pulsatile pseudoaneurysm on the posterolateral aspect at the mid femur was found. A sharp bone fragment was noted next to the pseudoaneurysm. The pseudoaneurysm was repaired by a vascular surgeon, and the bone fragment was removed. Following this procedure, the patient developed a subsequent periprosthetic joint infection requiring a double DAIR procedure six weeks following the pseudoaneurysm repair and is now on chronic antibiotic suppression. Orthopedic surgeons should be aware of the potential for pseudoaneurysm in the setting of total joint arthroplasty when treating a postsurgical hematoma of sudden onset.

## 1. Introduction

Total hip arthroplasty (THA) is an effective treatment for a wide range of conditions affecting the hip joint, and is commonly performed in the United States with an estimated 2.5 million individuals in the United States living with a total hip replacement [1, 2]. Vascular complications are among the most rare, albeit devastating, complications associated with THA, with an estimated 0.08–0.3% rate of occurrence [3]. Moreover, pseudoaneurysm formation is a rare subset of vascular injury, making this an extremely uncommon finding in THA [4].

A pseudoaneurysm is a collection of blood that collects between the tunica media and the tunica adventitia, the outer two layers of blood vessels, and is often due to continuous erosion of the blood vessel wall [5]. In the setting of THA, pseudoaneurysms can be difficult to diagnose, as clinical symptoms mimic symptoms of other more common complications, such as periprosthetic joint infection, hematoma, and nerve damage [6, 7].

We present a case in which a patient presented with symptoms of periprosthetic joint infection and hematoma. Upon return to the operating room for hematoma extraction, no overt signs of joint infection were identified, but a pseudoaneurysm was discovered. The patient initially recovered without complication after repair of the pseudoaneurysm and removal of the hematoma and bone fragment. Six weeks following this surgery, the patient developed a subsequent periprosthetic joint infection requiring a double debridement, antibiotics, and implant retention (DAIR) hip revision [8]. Our case demonstrates the importance of considering a pseudoaneurysm in the setting of hematoma and periprosthetic joint infection.

## 2. Case

**Figure 1:**
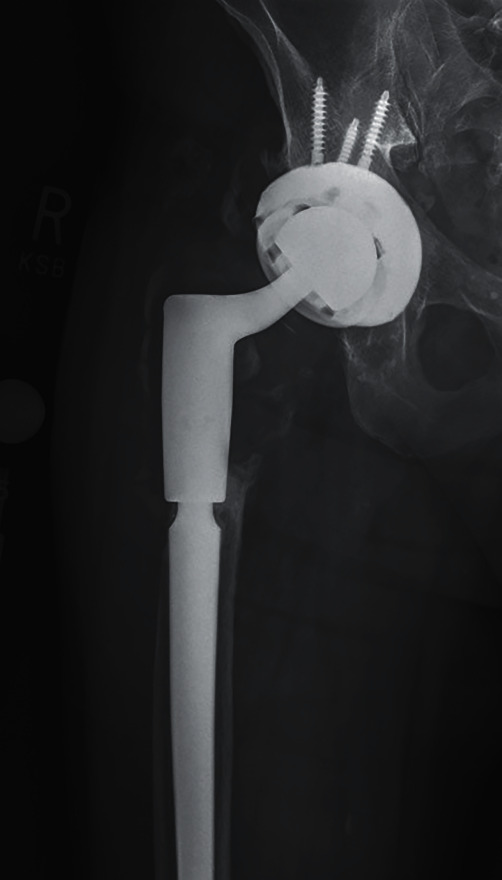
Preoperative anteroposterior X-ray of the right hip.

**Figure 2:**
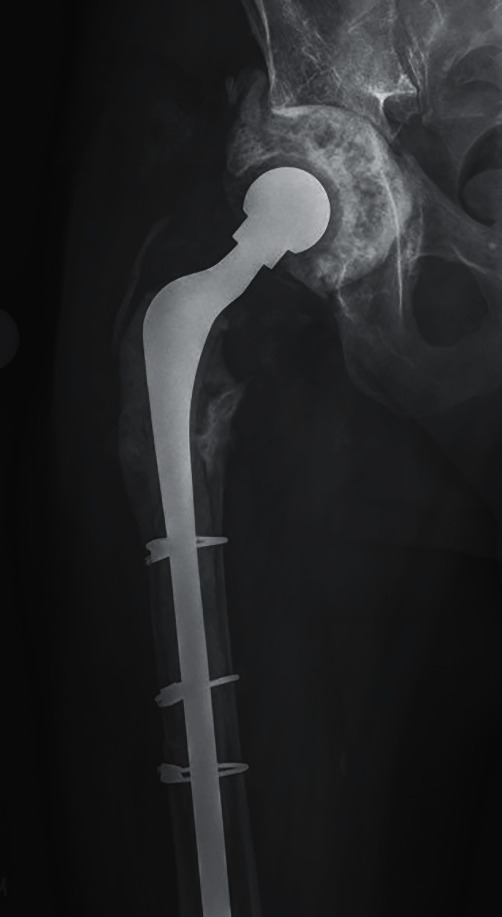
Anteroposterior X-ray of the right hip with antibiotic spacers.

**Figure 3:**
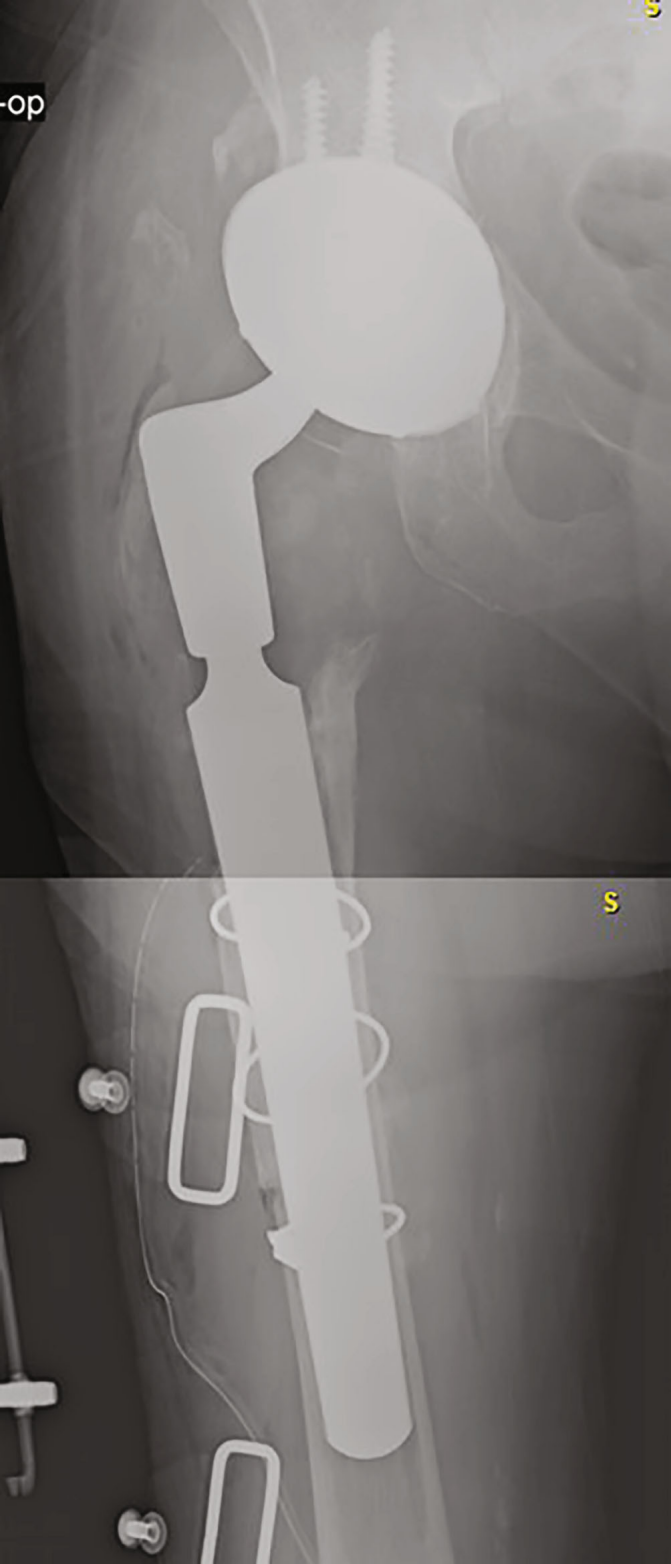
Anteroposterior X-ray of the right hip after reimplantation before fixation.

**Figure 4:**
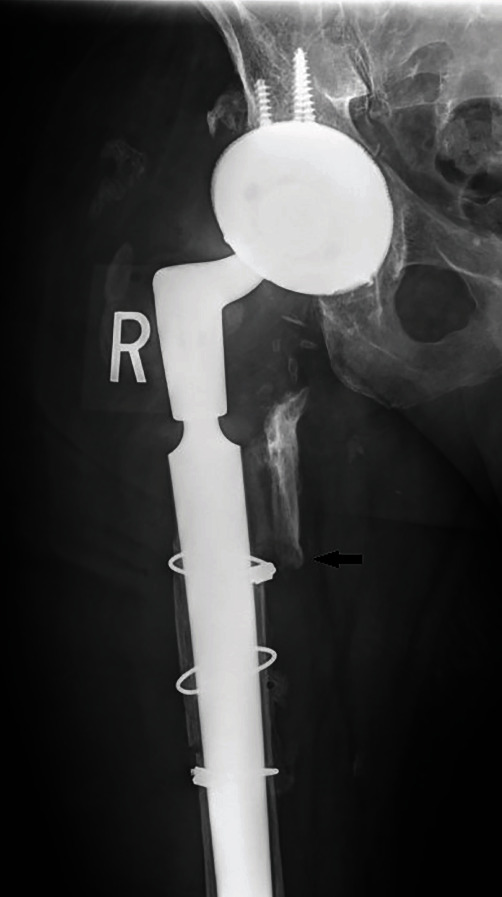
Anteroposterior X-ray of the right hip after reimplantation with fixation.

We present a 69-year-old male with a past medical history of atrial fibrillation with rapid ventricular rate and chronic opioid use. The patient's surgical history is notable for a slipped capital femoral epiphysis 56 years prior with subsequent right THA, multiple dislocations, and recurrent periprosthetic joint infection of the right hip. This patient presented to the clinic in May 2019 with right thigh pain with activity (Figure 1). A complete blood count and hip aspiration revealed a positive alpha defensin, positive *Streptococcus bovis* culture, elevated erythrocyte sedimentation rate (ESR) of 40 mm/hour, and elevated C-reactive protein (CRP) of 39 mg/L. The patient was referred to gastroenterology for possible colorectal cancer. Following this workup, the patient underwent a two-stage debridement with antibiotic spacer placement to treat the infected right hip joint. The first stage was performed in October 2019 (Figure 2). The patient was discharged to a skilled nursing facility seven days after the operations with a hip abduction brace and intravenous Ceftriaxone for six weeks. Three months later, resolution of the infection was confirmed with negative imaging findings, ESR of 17 mm/hour, CRP of 7.8 mg/L, synovial cell count of 852, negative leukocyte esterase, negative alpha defensins, and negative bacterial cultures (Figure 3). The second stage revision was performed in January 2020, and the patient was discharge home in 4 days after the operation with a stable hemoglobin after an uneventful hospital course (Figure 4).

Two weeks following the second stage, the patient presented to an outside facility with uncontrolled atrial fibrillation with rapid ventricular response and a sudden drop in hemoglobin to <7.0. The patient was transfused with 5 units of packed red blood cells, and his hemoglobin stabilized. Blood cultures were negative, and the orthopedic consultant had no concern for wound dehiscence or surgical site infection but noted mild acute swelling in the thigh. The patient was placed on prophylactic vancomycin and cefepime, and he was transferred to our institution upon stabilization of hemoglobin and resolution of atrial fibrillation.

**Figure 5:**
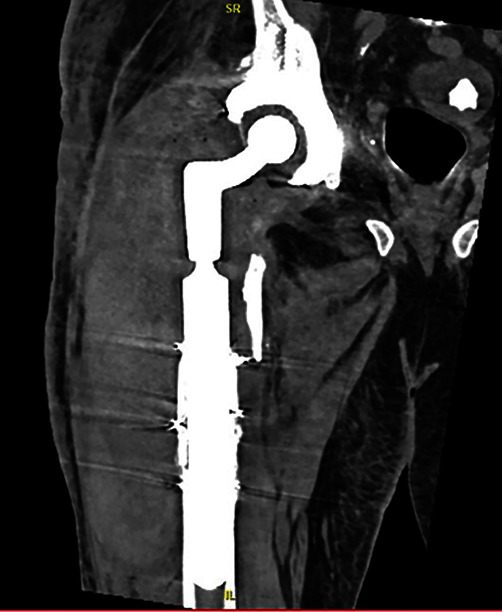
Computed tomography scan for right thigh swelling, revealing a 11.5 cm anteroposterior × 11 cm mediolateral × 33 cm craniocaudal hematoma.

**Figure 6:**
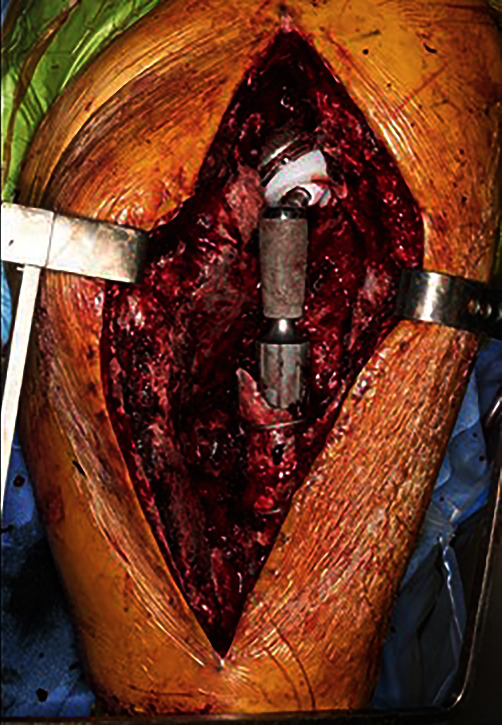
Pseudoaneurysm appreciated on the posterolateral aspect of the femur.

**Figure 7:**
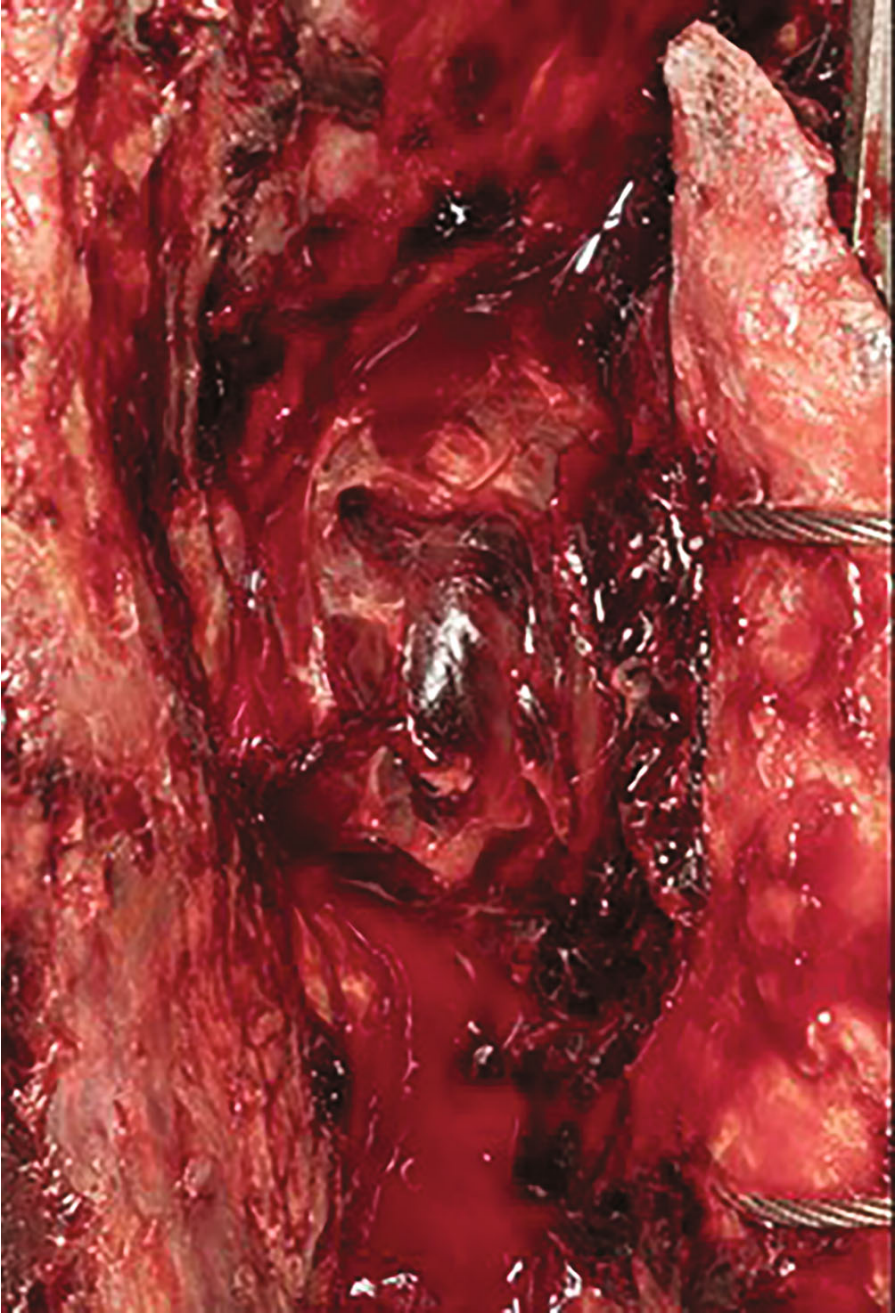
A close-up image of the pseudoaneurysm from Figure 6.

**Figure 8:**
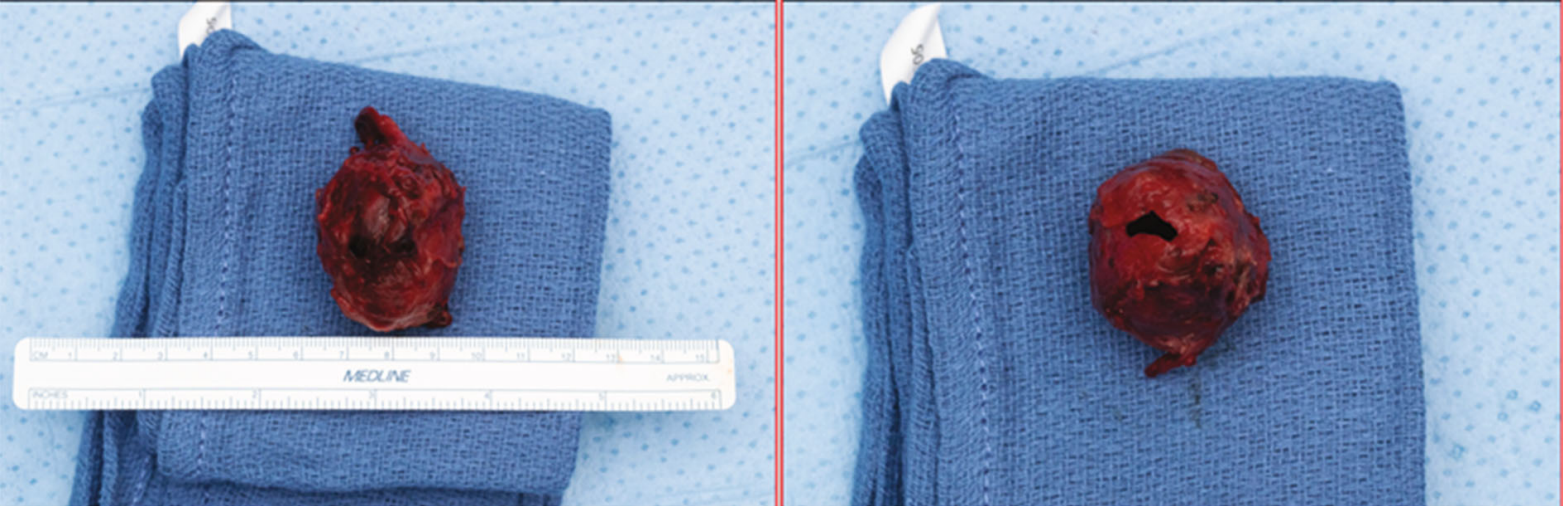
A bone fragment found in close proximity to the pseudoaneurysm.

Upon physical examination, severe swelling was noted of the right thigh. The patient's ESR and CRP levels were elevated at 45 mm/hour and 123 mg/L, respectively. Although ESR and CRP are of limited utility in the immediate postoperative period, given the patients history of recurrent infection, the authors had an elevated suspicion for possible reinfection. A computed tomography (CT) scan revealed a large hematoma 11.5 cm anteroposterior × 11 cm mediolateral × 33 cm craniocaudal on the lateral thigh (Figure 5). Two days later, the patient returned to the operating room for hematoma evacuation with irrigation and debridement. During hematoma evacuation, a large 3 cm × 5 cm × 3 cm pulsatile mass was found at the posterolateral aspect of the implant bone interface of the proximal femur (Figures 6 and 7). A vascular surgeon was consulted intraoperatively to assess and repair what was deemed intraoperatively as a pseudoaneurysm. Additionally, a sharp bone fragment (Figure 8) was found in close proximity to the pseudoaneurysm. This was removed at the time of the surgery. Postoperatively, the patient was given 30 mg daily subcutaneous Lovenox for three weeks based on the postoperative anticoagulation recommendations of the vascular surgery team, and he was made toe-touch weight bearing in an abduction brace. Standard antibiotics were administered for 24 hours postoperatively. The patient was discharged home on postoperative day 4 with two weeks of oral antibiotics.

At the patient's six-week postoperative visit following hematoma evacuation and pseudoaneurysm repair, the patient reported wound drainage and was subsequently found to have a recurrent periprosthetic joint infection of the right hip and underwent another a double DAIR procedure [8]. The patient is now on chronic antibiotic suppression and continues to suffer from right hip pain after these multiple revisions, necessitating the use of a walker and medical treatment for his chronic pain.

## 3. Discussion

Pseudoaneurysm formation following THA is a rare complication [9]. Although rare, it is more common in the revision setting when compared to the primary THA setting [10]. Most reported cases have a similar presentation of a quiescent period of several weeks up to 15 years followed by sudden onset of pain, swelling of the thigh, significant drop in hemoglobin, and cardiac instability [11]. Cement spicules and errant retractor placement are the most common reported causes of pseudoaneurysms in THA [12, 13].

A literature review performed by Pecoraro et al., examining 15 cases of deep femoral artery pseudoaneurysms in the setting of THA, revealed one case from a bone fragment and one case from a cement fragment that migrated from the acetabular component [11]. In our case study, we believe the pseudoaneurysm formation was likely a result of the sharp bone fragment discovered upon surgical excision of the hematoma (Figure 4 with arrow pointing to bone fragment) as this sharp fragment that likely resulted in injury to the blood vessel wall and subsequent aneurysm formation [5].

As vascular injury can result in serious complications, such as amputation, prosthetic joint infection (as seen on this case), shock, and mortality; early recognition with subsequent endovascular procedures is crucial for optimal outcomes for patients with a pseudoaneurysm [14, 15]. Distinguishing between a pseudoaneurysm and other more prevalent THA complications is challenging, as their clinical manifestations are shared by other common THA complications, such as periprosthetic joint infection, hematoma, and compartment syndrome. Furthermore, diagnosis is difficult as pseudoaneurysms are often located deep in the thigh and present with intact distal pulses making physical examination unreliable. Additionally, the infrequency of vascular complications during THA may make their diagnosis and treatment even more challenging [3, 16].

If a pseudoaneurysm is suspected, the authors recommend first line diagnostic imaging with ultrasound (US). This is a non-invasive, inexpensive, sensitive screening tool that can be used to visualize the vasculature of deep soft tissues. Duplex US has a sensitivity as high as 94% for the diagnosis of pseudoaneurysm [17]. US also decreases exposure to ionizing radiation and contrast dyes. Diagnostic challenges with US are operator dependent. If the US is inconclusive, a CT angiography should be obtained to rule out a pseudoaneurysm. CT angiography is the gold standard for pseudoaneurysm detection and is not operator dependent. CT angiograms can be obtained in minutes and have sensitivities as high as 95% with specificities as high as 100% [18, 19]. If a pseudoaneurysm is discovered, several treatment options exist including observation, compression therapy, endovascular interventions, and open repair. As the pseudoaneurysm was discovered incidentally during open surgery, we elected to perform an open aneurysm repair under direct visualization using Prolene sutures, which was without recurrence of pseudoaneurysm. Unfortunately, the patient suffered from a subsequent periprosthetic infection six weeks after pseudoaneurysm removal and hematoma evacuation. It is important to note that hematoma has been found to be associated with periprosthetic joint infection [20]. Successful treatments of pseudoaneurysms are traditionally carried out by with compression and/or thrombin injection. This approach has the benefit of minimal patient discomfort, high efficacy, and ability to use with anticoagulation. Reported success rates are as high as 93–100% with low complication rates [21, 22]. In patients who fail compression and US-guided thrombin injection, endovascular stent grafts and coil embolization may be used. Often this technique is inferior in terms of outcomes and cost when compared to compression and thrombin injection, and may represent a good option in patients who are not good candidates for open surgery [23]. These treatment modalities are much less invasive than open aneurysm repair but require pseudoaneurysm identification prior to open surgery. There are several factors that play into choosing the best treatment strategy for each patient. We recommend close communication with the vascular surgery team to arrive that the appropriate treatment for each individual patient.

Although the risk of vascular injury from THA is low, the mortality risk is high, with estimates reaching as high as 44% [6]. We advise surgeons to exercise extreme caution when treating patients with potential pseudoaneurysm formation and to consult a vascular surgeon for treatment when possible. In this case, the pseudoaneurysm was discovered incidentally during a planned hematoma removal, and repair was surgically indicated. In other cases where pseudoaneurysm is confirmed before an operation, informed consent regarding the treatment options, complications, and benefits should be obtained. Appropriate precautions during surgery, prompt recognition, and effective treatment can help reduce the mortality rate of pseudoaneurysm formation in the future.
